# Long-Term Impact of Hepatitis C Virus Eradication on Liver Stiffness in Egyptian Patients

**DOI:** 10.1155/2021/4961919

**Published:** 2021-09-20

**Authors:** Talaat Zakareya, Mostafa Elhelbawy, Hassan Elzohry, Mohamed Eltabbakh, Mohamed Deif, Mohamed Abbasy

**Affiliations:** ^1^Hepatology and Gastroenterology Department, National Liver Institute, Menoufia University, Shebeen El-Kom, Egypt; ^2^Radiology Department, National Liver Institute, Menoufia University, Shebeen El-Kom, Egypt

## Abstract

**Methods:**

Liver stiffness measurements (LSM) have been serially assessed 1, 3, and 5 years after HCV clearance in 655 patients who have been treated with DAAs.

**Results:**

The mean age was 51.44 ± 10 years. 73% of patients were males. 48% were cirrhotics. In noncirrhotics, the mean LSM was significantly decreased from 8.29 ± 2.3 kPa to 4.03 ± 1.0 kPa (*p* < 0.0001) at the end of the follow-up. Likewise, LSM decreased in cirrhotics from 29.66 ± 14.25 kPa to 22.50 ± 11.16 kPa (*p* < 0.0001). The proportions of *F*1, *F*2, *F*3, and *F*4 patients at the baseline were 17.7%, 17.9%, 16.6%, and 47.8%, which became 56.5%, 4.1%, 4.9%, and 34.5%, respectively, with a substantial reversal of cirrhosis in 87 patients (27.7%) at the end of follow-up.

**Conclusions:**

There was an overall significant regression of liver stiffness in all patients after sustained HCV eradication. Liver stiffness reflecting mild fibrosis (*F*0–*F*2) usually improves shortly after treatment, while measurements reflecting advanced fibrosis (*F*3–*F*4) take a longer time to regress to lower fibrosis stages.

## 1. Introduction

Hepatitis C virus (HCV) infection is a major worldwide health problem. Around 70% of persons acutely infected with HCV will develop chronic HCV infection. The global estimate of chronic HCV is about 71 million people (1% of the world population). The progression of hepatic fibrosis with excess deposition of an extracellular matrix is the most serious consequence of chronic hepatitis if left untreated. 15–30% of those with chronic liver injury will ultimately end in liver cirrhosis within 20 years with risk of cirrhosis-related complications including portal hypertension and hepatocellular carcinoma (HCC). The overall HCV related mortality is about 400,000 deaths every year [1, 2].

HCV treatment has been revolutionized since the introduction of direct acting antiviral agents (DAAs) in 2014. DAAs have been associated with high rates of sustained virological response (SVR) exceeding 95% with an excellent safety and tolerability profile [3].

However, the primary goal of HCV therapy is to achieve SVR, and the improvement of liver fibrosis remains the most important prognostic indicator. Therefore, it is necessary to determine how far virological clearance is associated with fibrosis regression [4].

Transient elastography is the most widely validated and approved noninvasive technique used to assess liver fibrosis with adequate accuracy and reproducibility. Although liver biopsy is the gold standard for the evaluation of liver fibrosis, it has been gradually replaced by TE because of its potential complications (pain, bleeding, etc.) and poor tolerability, particularly if serially required [5–10].

In many recent studies [11–19], liver fibrosis showed significant improvement shortly after DAAs, yet, to our knowledge, long-term effects on liver fibrosis are not fully investigated and addressed only in few cohorts.

In the current study, we aimed to evaluate the liver stiffness serially over 5 years after the successful eradication of HCV among Egyptian patients treated with DAAs.

## 2. Methods

The current study was conducted on 703 patients with chronic HCV who were treated with different interferon-free regimens of DAAs and successfully achieved SVR. Patients were recruited from hepatitis C virology clinic, national liver institute, Egypt. A written informed consent was obtained from each patient before inclusion. The study protocol complied with the ethical principles of Declaration of Helsinki (1975) and has been approved by the Local Institutional Review Board of National Liver Institute, Menoufia University.

The following categories of patients have been ruled out: cirrhosis with Child–Pugh score more than 7, HBV, autoimmune hepatitis, chronic kidney disease with eGRF <30 ml/min/1.73 m^2^, body mass index (BMI) of more than 32, alcohol abuse, HCC, extrahepatic malignancy, immunosuppressive therapy, solid organ transplantation, and poorly controlled diabetes mellitus (HbA1c more than 9%).

Potential concurrent etiologies of chronic liver disease were excluded through a panel of investigations including HBs Ag, anti-HBc, and autoimmune profile (total IgG and autoimmune markers including antinuclear antibodies (ANA), anti-smooth muscle antibodies (SMA), and anti-liver kidney microsomal antibodies (anti-LKM) [20, 21].

DAA regimens were given according to the recommendations of the European Association for the Study of the Liver (EASL, 2014) and the protocol approved by the Egyptian National Committee for the Control of Viral Hepatitis (NCCVH), 2015 [22, 23]: sofosbuvir (SOF) 400 mg/day plus ribavirin (weight based; 1200 mg if ≥75 kg or 1000 mg if <75 kg) for 24 weeks, sofosbuvir plus simeprevir (SIM) 150 mg/day for 12 weeks, sofosbuvir plus daclatasvir (DCV) 60 mg/day for 12 weeks in noncirrhotics, and SOF plus DCV plus ribavirin for 12 weeks in cirrhotics.

Sustained virological clearance was considered when HCV RNA becomes undetectable at week 12 after the completion of DAA therapy (SVR-12).

Patients have been prospectively followed for changes in liver stiffness using the Fibroscan 502 device (Echosens, Paris, France) at the time of achieving sustained virological clearance (SVR-12) and then at 1, 3, and 5 years. The standard M probe was basically used. Readings were expressed in kilopascals (kPa), and those with a success rate (the number of valid acquisitions divided by the number of attempts) more than 60% and interquartile range less than 30% were only considered. Liver stiffness measurement (LSM) represents the median of at least 10 valid measurements.

While the patient is lying in the dorsal decubitus with the right arm maximally abducted, acquisitions were applied through the intercostal spaces. The tip of the probe was covered with a lubricant gel before being applied to the intercostal skin. The selected liver portion to apply acquisitions had been estimated by the operator to be at least 6 cm thick and free of large vascular structures. The software determined whether each measurement was successful or not. Nonsignificant fibrosis (*F*0-1) was considered when LSM is less than 7.1 kPa, while *F*2, *F*3, and *F*4 (cirrhosis) were considered when LSM is (7.1 − 9.4), (9.5 − 12.4), and (≥12.5) kPa, respectively [24].

Values of liver stiffness measured at time points of the follow-up had been statistically compared with each other and with the baseline values reported just before the initiation of DAA therapy.

## 3. Statistical Analysis

Statistical analysis was performed using SPSS, version 22.0 for Windows (IBM Corp, Armonk, NY, USA). Graphical illustrations were created using Microsoft Excel 2013. The descriptive statistics for quantitative variables were presented as mean and SD, while qualitative variables were presented as numbers and percent. The comparison of qualitative data was performed using the chi-square test or Fisher's exact test, where appropriate. The continuous variables across time were compared using the paired *t*-test or the Wilcoxon signed-rank test. Mann–Whitney, Kruskal–Wallis, and Friedman tests were used for nonparametric data. ANOVA statistics with posthoc analysis were used to verify statistical significance of LSM at different time points of follow-up. Statistical analysis was considered significant when the *p* value was less than 0.05.

## 4. Results

**Figure 1:**
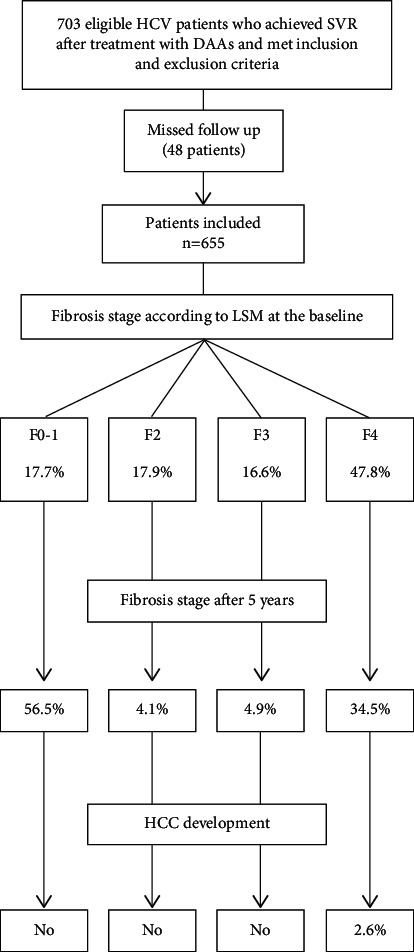
Flowchart of the study showing the proportion of each fibrosis stage at the baseline and at the end of the study and the rate of de novo HCC. SVR, sustained virological response; DAAs, direct antiviral agents; HCC, hepatocellular carcinoma.

Among the initially eligible patients who fulfill the inclusion and exclusion criteria, missed follow-up has been reported in 48 patients. Therefore, the ultimate number of included patients who completed the follow-up and underwent final analysis was 655 patients (Figure 1). The baseline demographic, clinical, and laboratory data are shown in Table 1. The mean age was 51.44 ± 10 years, and 73% of patients were males. Based on the baseline LSM, patients were categorized into cirrhotics and noncirrhotics. 313 patients (47.8%) had LSM consistent with liver cirrhosis (≥12.5 kPa). The remaining patients were noncirrhotics with LSM <12.5 kPa. Noncirrhotics were subdivided into 109 patients (16.6%) with advanced fibrosis (*F*3), 117 patients (17.9%) with mild fibrosis (*F*2), and 116 patients (17.7%) with minimal or insignificant fibrosis (F0-1). 66.4% of the cirrhotic patients were Child–Pugh class A. The rest were Child–Pugh class B (33.6%). The mean LSM was 29.66 ± 14.25 kPa in cirrhotics and 8.29 ± 2.31 kPa in noncirrhotics.

In general, there were significant serial decline in LSM in all patients at each time point of the follow-up along the course of the study (*p* < 0.0001). In noncirrhotics, LSM steadily decreased over time where it became 4.03 ± 1.0 kPa (*p* < 0.0001) at the 5th year with a 51.4% decrease as compared with the baseline value. In the same stream, LSM significantly declined in cirrhotic patients till it became 22.50 ± 11.16 kPa at the 5th year with a 24.1% decrease as compared with the baseline LSM. Although LSM decreased significantly in both noncirrhotic and cirrhotic patients, the rate of decline was greater, faster, and more significant in noncirrhotics than in cirrhotics at the earlier time points of the follow-up (at SVR and 1 year). Beyond the first year, cirrhotic patients attained more prominent trends of decline till the end of the follow-up. The detailed ANOVA statistics and post hoc analysis are shown in Table 2.

In noncirrhotics, the mean decline in LSM, as compared with the baseline values, was 2.4, 3.4, 4, and 4.3 kPa at SVR, 1, 3, and 5 years, respectively. On the other hand, it was 1.3, 3.8, 5.9, and 7.2 kPa, consecutively, in cirrhotic patients. Detailed comparative statistics between mean difference changes in LSM at each time point and other time points are shown in Table 3. Notably, almost all comparisons were highly significant in noncirrhotic patients (*p* < 0.0001). Unlikely, high significant comparisons in cirrhotic patients were only noted when comparing measurements at relatively remote time points, i.e., LSM at baseline with LSM at 3 and 5 years and LSM at SVR versus LSM at 3 and 5 years.

**Figure 2:**
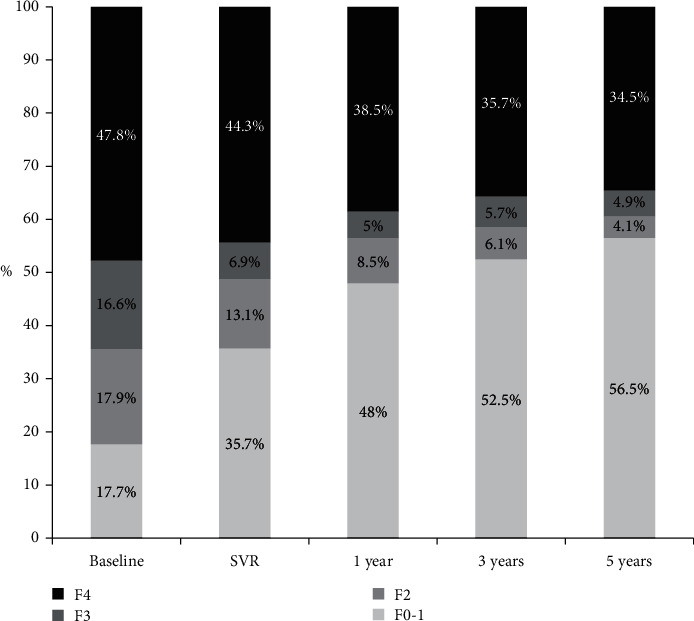
Proportions of fibrosis stages at different time points of follow-up. SVR, sustained virological response.

The changes in the proportions of each fibrosis stage in the studied patients at each time point of the follow-up are shown in (Figure 2).

The number of cirrhotic patients at the baseline was 313. This number decreased to 226 at the end of the follow-up. This means that cirrhosis has regressed to lower fibrosis stages in 87 patients (27.7%).

The ratio of *F*3 patients has regressed from 16.6% at the baseline to 4.9% at the end of the follow-up with transition to lower fibrosis stages in 70.5% of these patients. Regression to lower fibrosis stages was more prominent in *F*2 patients where 77.1% of these patients have regressed to *F*0-*F*1.

As being noted, the incremental increase in the ratio of F0-1 over the follow-up period from 17.7% at the baseline to 56.5% came in parallel with regression in the proportions of *F*4, *F*3, and *F*2. This could be referred to HCV eradication; the basic underlying etiology of liver fibrosis and lack of any additive liver insult.

Noteworthy, we did not report any increase in LSM in patients with minimal (*F*0-*F*1) and mild fibrosis (*F*2), while trivial insignificant progression (1.7 ± 0.2 kPa, *p*=0.17) has been reported in only 5 patients (4.6%) with *F*3 fibrosis at the end of follow-up. Yet, none of these patients has progressed to *F*4. These patients remained stable with no clinical complications till the end of the study.

Among cirrhotic patients, 28 (8.9%) had a nonsignificant increase in LSM (2.1 ± 0.3 kPa, *p*=0.09) at the end of the follow-up. Of these patients, 6 had clinical and laboratory deterioration, 5 of them died, and 1 received liver transplantation. Two patients developed HCC. The remaining 20 patients were clinically stable till the end of the follow-up without any features of liver decompensation.

HCC developed in 6 cirrhotic patients without any clinical and/or laboratory deterioration. Surprisingly, these patients had a regressive pattern of LSM. There were no reported cases of HCC among noncirrhotic patients.

Therefore, HCC has developed in 8 cirrhotic patients (2.6%) (2 of them had a progressive pattern of LSM, while the remaining 6 had a regressive pattern). Of those patients, 5 successfully managed with locoregional therapy, and 3 passed away due to aggressive and infiltrative tumor associated with rapid clinical deterioration.

The overall mortality has been reported in 11 patients (1.65%): 5 due to liver deterioration and failure, 3 due to aggressive HCC, and 3 due to non-liver-related causes (2 cardiopulmonary diseases and 1 cerebrovascular stroke).

## 5. Discussion

HCV is a major leading cause of liver cirrhosis and its related complications. The annual rates of liver decompensation, transplantation, and HCC attributed to HCV are 6.37%, 4.58%, and 3.36%, respectively [25].

Egypt has the highest HCV burden in the world [26–28]. Since the introduction of DAAs, the Egyptian government has initiated a large campaign under supervision of NCCVH for mass screening and treatment of HCV and put 2030 as an expected end point to announce “Egypt free of HCV.” In the period between 2014 and 2018, about 1.8 million patients have been successfully treated [29].

Despite this big achievement in the primary goal of treatment, which is viral clearance, we have no confirmed data about the secondary goal, which is minimizing or preventing complications. Doubtless, this secondary goal is closely reflected by the regression of liver fibrosis. Unfortunately, we have no definitive data about long-term changes in liver fibrosis, particularly in patients with advanced fibrosis and cirrhosis, after successful eradication of HCV. This was the basic motivation to perform the current study.

In the view of the available literature, we will discuss the principal findings of the present study. Globally, there was a significant decrease in LSM in noncirrhotics (*p* < 0.001). Furthermore, posthoc analysis revealed a significant decline when comparing LSM at any given time point with other time points of the follow-up except when comparing LSM at the 4th and 5th years (*p*=0.44). The mean overall decline in LSM in those patients at the end of the follow-up was 4.26 kPa, representing about 51% of the baseline value (4.03 ± 1.0 versus 8.29 ± 2.31). Notably, 78.6% (3.35 kPa) of this decline has been achieved in the first year posttreatment (4.94 ± 1.89 versus 8.29 ± 2.31). This means that liver fibrosis, whenever frank cirrhosis is not reached, could be resolved shortly after successful treatment.

The rapid fall in LSM, observed shortly after treatment, has been described in many studies [1–4].

In addition, there was an overall significant decline in LSM among cirrhotic patients (*p* < 0.0001). However, the posthoc analysis was statistically nonsignificant when comparing LSM at each two successive time points (e.g., baseline vs. SVR and SVR vs. 1 y,…, etc.), while it was significant when comparing LSM at nonsuccessive and remote time points (i.e., baseline vs. 1 y, 3 y, and 5 y, SVR vs. 3 y and 5 y, and 1 y vs. 5). This means that changes in LSM, in those patients, take longer time to attain a statistically significant value. Therefore, a long-term follow-up of liver stiffness, when intended, should be optimized to be at longer intervals of at least 3 years. However, this should not defer regular periodic HCC surveillance as these patients are still at a risk of developing HCC.

In cirrhotics, there was an average drop of 7.16 kPa in LSM by the end of the follow-up representing 24% of the baseline value. 52.5% of this decrease (3.76 kPa) has been achieved in the first year, 27.6% (2.1 kPa) in the 3rd year, and 17.1% (1.3 kPa) in the 5th year. It is worthy to note that the overall decline in LSM over 5 years was 24% of the baseline value. This indicates that the extracellular matrix (ECM) in those patients is more compact with excess interlacing fibers, which need longer time to be degraded. The actual time required for complete degradation is not yet established. However, undoubtedly the higher the baseline LSM, the longer the time required for the degradation of ECM.

Furthermore, with that reported decline in LSM of 7.16 kPa, we could expect the reversal of cirrhosis in patients with baseline LSM of less than 20 kPa within 5 years after successful treatment.

Data from many studies with a relatively longer follow-up came in agreement with our findings; first, a German study by Pietsch et al., LSM has significantly decreased from 13.1 to 7.9 kPa (*p* < 0.0001) over a follow-up of 96 weeks [30]. Second, a study by Mandofer et al. [31] revealed a significant decrease in LSM (3.6 kPa, *p* < 0.001) 48 weeks after treatment. However, this study was conducted on HCV/HIV coinfected patients, and 52% of them have undergone treatment. Additionally, the sample size was so limited (31 patients). Another cohort by Bachofner et al. [12] conducted on 392 patients revealed a significant decrease in the median of LSM from 12.65 to 8.55 kPa (*p* < 0.001) within 18 months after DAA treatment representing 34.2% decline in the baseline LSM; however, this study was limited by being retrospective.

Changes in the ratios representing different stages of fibrosis along the follow-up were notable and favorable. LSM concordant to *F* (0-1), *F*2, and *F*3 at the baseline were 17.7%, 17.9%, and 16.6%, respectively. These ratios became 56.5%, 4.1%, and 4.9% at the 5th year, respectively. The achieved regression in liver stiffness has been reflected as increased ratios of lower fibrosis stages at the expense of ratios of higher fibrosis stages.

In the same stream, the number of *F*4 patients at baseline were 313 (47.8%) and became 226 (34.5%) at the 5th year, which means that cirrhosis has been regressed to lower stages of fibrosis in 87 patients (27.8%). In a cohort conducted on 304 patients treated with DAAs, there was about 20% decrease in the proportion of *F*4 patients (*p* < 0.0001) at 24 weeks after treatment [18]. Our results at 1 year posttreatment are comparable with these rates where the number of F4 patients has regressed to 252 representing 19.5% drop in *F*4 patients.

An Egyptian study conducted by Shiha et al. [32] showed the reversal of cirrhosis in 21.8% of *F*4 patients over a follow-up of 2 years. These findings are close to what we found at 3 years, where cirrhosis has been reversed in 234 (25.2%) patients. In addition, they reported progression from *F*3 to *F*4 in 11.4% of patients. This is inconsistent with our results where we reported a trivial progression of LSM in only 4.6% of *F*3 patients without transition to *F*4. These differences may be referred to variability in the sample size (*F*3 patients were 631 in the Shiha study versus 109 in the current study). In addition, the reference range of LSM for each fibrosis stage was quite different in both studies (*F*3 was 10.2 − 16.3 kPa in Shiha cohort versus 9.5 − 12.4 kPa in the current study while *F*4 was >16.3 kPa versus >12.4 kPa in our study). In addition, Pietsch et al. reported a higher rate (17%) of fibrosis progression over a follow-up of 96 weeks. However, this study was limited by the relatively small sample size (143 patients). In addition, the increase in LSM was nonlinear with an initial decrease at week 24 followed by an increase between weeks 24 and 96, and the authors could not provide a convincing explanation for this [30].

In the current study, we would like to clarify that we have reported an unexpected increase in liver stiffness in 28 patients among sustained responders. This increase was in cirrhotics only. Although this increase was statistically insignificant (*p*=0.09), we could not find an explanation for this, particularly, since we had excluded all major possible concomitant causes that could affect the liver, basically HBV, autoimmune hepatitis, hepatotoxic drugs, and alcohol abuse. In addition, patients with a raised suspicion of nonalcoholic fatty liver disease (NAFLD) were also excluded, including obese patients and with poorly controlled diabetes. Surprisingly, BMI has decreased at the end of the study when compared with the baseline value (27.9 ± 1.5 kg/m^2^ versus 28.3 ± 1.4 kg/m^2^). Despite this, liver histology remains a mandatory requirement to exclude NAFLD, in view of a recent term of “lean NAFLD,” which indicates the possibility of NAFLD to occur in patients with normal BMI.

From our perspective, this issue remains an interesting point for research, and further large scale multicenter studies and extensive investigations, including liver pathology, are required to stand on the actual underlying hidden factors and operating mechanisms for this increase so as to appropriately manage those patients and avoid future complications.

The reported rate of HCC development after the treatment of HCV is quite variable among different studies as some of these reports came from retrospective single-center studies and due to variability in the sample size and duration of the follow-up periods. In addition, some of these studies included cirrhotic patients only, while others included both cirrhotic and noncirrhotic patients. Furthermore, some studies were carried out on responders only and others included all patients with or without SVR. [33–41]. In the study by Finkelmeier et al. [36], the rate of de novo HCC development in 819 patients after DAA therapy was 3.1%. 269 of the included patients were cirrhotics. The HCC rate was increased to 8.9% when calculated in the subcohort of cirrhotics. No HCC was reported in noncirrhotics. In addition, a large prospective study from France (7344 patients treated with DAAs versus 2551 untreated controls with a median follow-up of 33.4 months) reported that DAA treatment was associated with a reduced risk of developing HCC after the adjustment for other confounding variables (adjusted hazard Ratio 0.66, 95%CI 0.46 − 0.93). These findings were consistent with our results [37]. These findings were also confirmed by a German study on cirrhotic patients where a reduced 5 year HCC risk has been reported (2.04% in patients treated with DAAs versus 5.04% for untreated patients, *p*=0.008) [38]. In addition, data from a large Italian prospective study of 2,249 patients with HCV associated cirrhosis reported an incidence rate of HCC of 3.5% during a mean follow-up of 14 months (6–24 months). The absence of SVR was reported as a significant independent factor associated with an increased risk of HCC (HR = 3.40, 95% CI = 1.89 − 6.12, *p* < 0.001) [39]. Among 22,500 patients treated with DAAs, Kanwal et al. reported a significantly reduced risk of HCC in patients with SVR as compared with patients without SVR (0.90 vs. 3.45 HCC/100 person years; adjusted HR, 0.28, 95% CI = 0.22 − 0.36) [38]. Another large retrospective study from the US reported reduced short term HCC occurrence among cirrhotic patients with DAA-induced SVR (2.12%) and IFN-induced SVR group (2.28%) when compared with the untreated group (4.53%) [41].

All these reports were consistent with our results. In our study, the rate of HCC development was 1.2% of all patients and 2.6% of cirrhotic patients. This rate is quite lower than that reported in some of the previous studies. This difference could be attributed to the heterogeneity of inclusion criteria in our cohort and these studies where we selectively included sustained responders. In addition, cirrhotic patients with Child–Pugh score >7 (late class B and class C), who are more prone to develop HCC, were not included in our study. This could share in part the reported lower rate of de novo HCC among our patients.

Indeed, liver fibrosis is an important risk factor for HCC development, where patients with established cirrhosis are at a higher risk than those in lower fibrosis stages [42, 43]. The improvement of liver histology and regression of liver fibrosis associated with SVR is an important factor in lowering the rate of HCC occurrence [44]. This concept has been confirmed in our results.

One of the basic limitations of the present study was the inability to include patients with advanced and decompensated cirrhosis, whereas these patients were ineligible to receive the available DAAs at the time of performing the study. Another limitation is unfeasibility to perform paired histological assessment to verify and confirm that the improvement in LSM reflects a true reversal of fibrosis and regaining of normal lobular parenchymal architecture and does not represent a mere reflection of ameliorated liver inflammation.

Finally, we can conclude that HCV elimination after treatment with DAAs is generally associated with a significant regression of hepatic fibrosis. Early and mild fibrosis usually improves and could substantially vanish at earlier time; however, advanced fibrosis and cirrhosis take longer time periods to achieve significant improvement. In addition, we have to emphasize that successful viral clearance in cirrhotic patients does not preclude the risk for developing HCC and does not obviate the need for continued HCC surveillance.

## Figures and Tables

**Table 1:** Demographic and baseline data.

*n* = 655	Mean ± SD, *n* (%)
Demographics	
Age (years)	51.44 ± 10.45

Sex, *n* (%)	
Male	478 (73%)
Female	177 (27%)
BMI	29.13 ± 2.80
Diabetes mellitus	180 (27.4)
Hypertension	87 (13.3)

Laboratory data	
Albumin (gm/dL)	4.08 ± 0.54
Bilirubin (mg/dL)	0.83 ± 0.45
INR	1.11 ± 0.13
ALT (IU/l)	65.10 ± 51.89
AST (IU/l)	88.98 ± 39.60
Hb (gm/dl)	14.08 ± 1.77
WBCs (×10³/mm³)	6.35 ± 2.12
Platelets (×10³/mm³)	178.17 ± 71.63
HCV RNA (IU/ml)	1526115.6 ± 2650113
Fasting blood glucose (mg/dL)	112 ± 55

Baseline LSM (kPa)	18.4 ± 14.45
Noncirrhotics	8.29 ± 2.31
Cirrhotics	29.66 ± 14.25

State of fibrosis according to LSM	
*F*0-1	116 (17.7%)
*F*2	117 (17.9%)
*F*3	109 (16.6%)
*F*4 (cirrhosis)	313 (47.8%)
Child–Pugh class A	208 (66.4%)
Child–Pugh class B	105 (33.6%)
Child–Pugh class C	0 (0%)

Treatment regimen, *n* (%)	
SOF + RBV	302 (46.1%)
SOF + SIM	28 (4.3%)
SOF + DCV ± RBV	325 (49.6%)

BMI, body mass index; ALT, alanine aminotransferase; AST, aspartate aminotransferase; Hb, hemoglobin; WBCs, white blood count; HCV RNA, hepatitis C virus ribonucleic acid; INR, international normalized ratio; kPa, kilo Pascal; LSM, liver stiffness measurement; SOF, sofosbuvir; RBV, ribavirin; SIM, simiprevir; DCV, daclatasvir.

**Table 2:** Comparison between mean LSM at different time points of follow-up.

LSM (kPa)	At baseline (1)	At SVR-12 (2)	At 1 year (3)	At 3 years (4)	At 5 years (5)	*F*	*p* value	Post hoc
All patients	18.47 ± 14.6	16.59 ± 15.01	14.92 ± 14.23	13.57 ± 13.67	12.8 ± 313.29	17.104	< 0.0001^*∗*^	All were < 0.0001^*∗*^, except
								1 versus 2 = 0.117
								2 versus 3 = 0.205
								2 versus 4 = 0.001^*∗*^
								3 versus 4 = 0.417
								3 versus 5 = 0.58
								4 versus 5 = 0.878

Noncirrhotics	8.29 ± 2.31	5.90 ± 2.30	4.94 ± 1.89	4.27 ± 1.25	4.03 ± 1.0	305.333	< 0.0001^*∗*^	All were < 0.0001^*∗*^, except
								4 versus 5 = 0.44

Cirrhotics	29.66 ± 14.25	28.36 ± 14.05	25.90 ± 13.03	23.80 ± 10.88	22.60 ± 10.06	14.360	< 0.0001^*∗*^	All were < 0.0001^*∗*^, except
								1 versus 2 = 0.774
								1 versus 3 = 0.007^*∗*^
								2 versus 3 = 0.181
								3 versus 4 = 0.332
								3 versus 5 = 0.02^*∗*^
								4 versus 5 = 0.774

kPa, kilo Pascal; LSM, liver stiffness measurement; SVR, sustained virological response. ^*∗*^Significant at the 0.05 level.

**Table 3:** Mean difference in LSM at different time points of follow-up.

Time point (1)	Time point (2)	All patients		Noncirrhotics		Cirrhotics	
		Mean difference (1 − 2)	*p*	Mean difference (1 − 2)	*p*	Mean difference (1 − 2)	*p*
SVR	Baseline	−1.87603	0.117	−2.40000^*∗*^	< 0.0001	−1.30000	0.774
1 year	Baseline	−3.54916^*∗*^	< 0.0001	−3.35510^*∗*^	< 0.0001	−3.76250^*∗*^	0.007
	SVR	−1.67313	0.205	−0.95510^*∗*^	< 0.0001	−2.46250	0.181

3 years	Baseline	−4.90244^*∗*^	< 0.0001	−4.02915^*∗*^	< 0.0001	−5.86250^*∗*^	< 0.0001
	SVR	−3.02641^*∗*^	0.001	−1.62915^*∗*^	< 0.0001	−4.56250^*∗*^	< 0.0001
	1 year	−1.35328	0.417	−0.67405^*∗*^	< 0.0001	−2.10000	0.332

5 years	Baseline	−5.64565^*∗*^	< 0.0001	−4.26589^*∗*^	< 0.0001	−7.16250^*∗*^	< 0.0001
	SVR	−3.76962^*∗*^	< 0.0001	−1.86589^*∗*^	< 0.0001	−5.86250^*∗*^	< 0.0001
	1 year	−2.09649	0.058	−0.91079^*∗*^	< 0.0001	−3.40000^*∗*^	0.021
	3 years	−0.74321	0.878	−0.23673	0.440	−1.30000	0.774

SVR, sustained virological response. ^*∗*^The mean difference is significant at the 0.05 level.

## Data Availability

Data used to support the findings of this study are available from the corresponding author upon request.
